# Development and Validation of an LC-MS/MS Method and Comparison with a GC-MS Method to Measure Phenytoin in Human Brain Dialysate, Blood, and Saliva

**DOI:** 10.1155/2018/8274131

**Published:** 2018-04-01

**Authors:** Raphael Hösli, Stefan König, Stefan F. Mühlebach

**Affiliations:** ^1^Clinical Pharmacy and Epidemiology, Hospital Pharmacy, University of Basel, Spitalstrasse 26, CH-4031 Basel, Switzerland; ^2^Spitalzentrum Biel, Apotheke, Vogelsang 84, CH-2501 Biel-Bienne, Switzerland; ^3^Division of Forensic Medicine, University of Bern, Bühlstrasse 20, CH-3012 Bern, Switzerland

## Abstract

Phenytoin (PHT) is one of the most often used critical dose drugs, where insufficient or excessive dosing can have severe consequences such as seizures or toxicity. Thus, the monitoring and precise measuring of PHT concentrations in patients is crucial. This study develops and validates an LC-MS/MS method for the measurement of phenytoin concentrations in different body compartments (i.e., human brain dialysate, blood, and saliva) and compares it with a formerly developed GC-MS method that measures PHT in the same biological matrices. The two methods are evaluated and compared based on their analytical performance, appropriateness to analyze human biological samples, including corresponding extraction and cleanup procedures, and their validation according to ISO 17025/FDA Guidance for Industry. The LC-MS/MS method showed a higher performance compared with the GC-MS method. The LC-MS/MS was more sensitive, needed a smaller sample volume (25 *µ*L) and less chemicals, was less time consuming (cleaning up, sample preparation, and analysis), and resulted in a better LOD (<1 ng/mL)/LOQ (10 ng/mL). The calibration curve of the LC-MS/MS method (10–2000 ng/mL) showed linearity over a larger range with correlation coefficients *r*^2^ > 0.995 for all tested matrices (blood, saliva, and dialysate). For larger sample numbers as in pharmacokinetic/pharmacodynamic studies and for bedside as well as routine analyses, the LC-MS/MS method offers significant advantages over the GC-MS method.

## 1. Introduction

Sensitive and specific quantification methods are of critical importance when monitoring individualized drug therapy in patients or investigating drug concentration in forensic toxicology [1]. Critical dose drugs but also newly developed and designed complex drugs require analytical methods to check for effective drug delivery to target tissues and to minimize toxicity in sensitive organs or cells. When such drugs have to be used in patients with varying pharmacokinetics (PK) (e.g., ICU patients), an appropriate therapeutic drug monitoring (TDM), which allows, for example, to correlate the drug concentration in easy accessible plasma samples with those in the tissue of action, becomes even more relevant for a safe and efficient drug treatment [2].

Phenytoin (PHT) belongs to the most widely prescribed drugs to prevent and control most types of seizure disorders and to treat epilepsy [3]. It is one of the most often used critical dose drugs where insufficient or excessive dosing can have severe consequences such as seizures or toxicity. Thus, the monitoring and precise measuring of PHT concentrations in patients is crucial [4, 5]. As an example, in forensic toxicology, epilepsy patients under PHT treatment who have been involved in an accident have to be analyzed in order to verify whether the PHT concentration was adequate or possibly the reason for the accident [6]. However, there are several characteristics of PHT including a relatively low therapeutic index, difficult pharmacokinetics (PK) and pharmacodynamics (PD), saturable oxidative biotransformation, and the nonlinear clearance, which complicate a therapeutic drug monitoring (TDM) aimed at preventing intoxication of patients or treatment failures [7].

Thus, researchers and practitioners are interested in specific, sensitive, robust, and cost-effective methods to identify PHT concentrations in patients. Thereby, several compartments to measure the PHT concentration could be addressed such as blood, saliva, and CNS fluid (microdialysate). The correlation of PHT in different body compartments is not yet completely understood and has only recently been addressed by researchers who have compared the measurement of PHT in these different compartments with a GC-MS method [8]. While the GC-MS has long been the standard method in forensic testing, LC-MS/MS methods have become more common, as they generally offer some advantages over GC-MS [9]. Recently, researchers have developed an LC-MS/MS method to measure PHT in one specific body compartment (i.e., blood plasma or serum) [10]. Missing, however, is a thorough comparison of the performance of these two analytical methods in the detection and analysis of PHT in different body compartments (i.e., blood, saliva, and samples from brain tissue microdialysis).

The aim of the present study was to develop and validate an LC-MS/MS method for the measurement of PHT concentrations in different body compartments such as blood and saliva, as well as samples from brain tissue microdialysis often used in neurology and neurosurgery, where antiepileptic therapy is often mandatory [11, 12], and to compare its efficiency with a formerly developed GC-MS method [8]. The fact that this established GC-MS method measured PHT in the same biological matrices (i.e., blood, saliva, and human brain dialysate) enables a reliable comparison with regard to the performance of GC-MS versus LC-MS/MS in measuring PHT in different body compartments. The two methods are evaluated and compared based on their analytical performance, appropriateness to analyze human biological samples, including corresponding extraction and cleanup procedures, and their validation according to ISO 17025/FDA Guidance for Industry [13]. Finally, the suitability of the two analytical methods for PK/PD studies, bedside measurement, and forensic use is discussed. In addition, the LC-MS/MS method developed in the current study is compared with an established LC-MS/MS method which measured PHT in blood plasma samples [10].

## 2. Materials and Method

### 2.1. Chemicals and Samples Used for the Development of the LC-MS/MS Method and Its Validation

PHT reference substance was purchased from Desitin Pharma GmbH (Liestal, Switzerland) and from the European Pharmacopoeia (PHT Ph. Eur. Standard, EDQM, Strasbourg, France). The IS for LC-MS/MS was PHT-D_10_ (PHT D-10, C_15_H_2_D_10_N_2_O_2_, MW = 262.33) in methanol (MeOH) (100 *μ*g/mL) from Cerilliant (Round Rock, TX).

Calcium chloride, perchloric acid, citric acid monohydrate, potassium chloride, magnesium chloride hexahydrate, sodium chloride, sodium hydroxide, and the solvents (methanol, acetic acid 100%, and acetone) were of analytical grade and purchased from Merck (Darmstadt, Germany). Artificial cerebrospinal fluid (aCSF; dialysate solution) was prepared according to M Dialysis AB (Stockholm, Sweden) [14]. Blood CPDA-1 (anticoagulant citrate phosphate dextrose adenine solution; to simplify only named blood in the following) was obtained from the Blood Donor Center (Bern, Switzerland). Saliva was obtained from one of the investigators. 20–60 *μ*L PHT-containing samples from patients collected from a 2 *µ*L/min flow rate brain microdialysis and 2 mL of CPDA containing PHT patient blood samples were provided by the Department of Neurosurgery (Kantonsspital Aarau AG, Switzerland and Centre Hospitalier Universitaire Vaudois, Switzerland). All biological samples (blood and dialysates) were frozen and stored at −24°C. Before sample analysis, the samples were thawed at room temperature for 30 minutes and homogenized by shaking with a vortex for one minute.

### 2.2. Internal Standards, Calibrator Standard System Suitability Testing, and Sample Preparation

The internal standard (IS) stock solution was prepared by adding 100 *µ*L of the PHT-D_10_ (100 *µ*g/mL) to 9900 *µ*L of MeOH. 5 mL of this solution was added to 95 mL of 1 M perchloric acid aqueous solution to get the final concentration of 50.0 ng/mL, which is used as IS working solution. The PHT reference stock solution (1.00 mg/mL) was used to obtain eight calibration (Cal) solutions with concentrations of 2000, 1000, 500, 250, 100, 50, 20, and 10 ng/mL PHT. 20 *µ*L of these Cal solutions were added to 980 *µ*L of the biomatrices to get the Cal working solutions. For quality control (QC), solutions with 1600, 400, 30, and 10 ng/mL PHT were prepared out of PHT reference stock solution (1.00 mg/mL).

**Figure 1:**
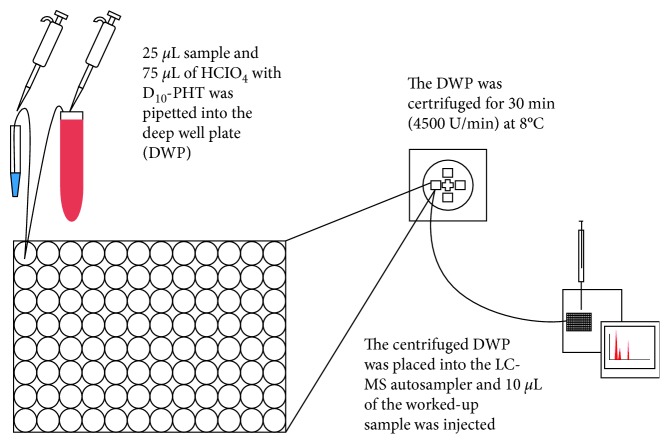
Sample preparation for the LC-MS/MS analyses for blood, saliva, and aCSF samples.

The IS working solution of 75 *µ*L was added either to an aliquot of 25 *µ*L Cal working solution, QC solutions, or 25 *µ*L sample from patients containing PHT. The sample preparation for the LC-MS/MS consisted of pipetting 75 *µ*L of IS working solution to 25 *µ*L sample into a deep well plate (0.6 mL, Chemie Brunschwig AG, Basel, Switzerland) covered by a sealing mat (Silicone, Chemie Brunschwig AG, Basel, Switzerland). The well plates were rigorously shaken for 5 minutes and then centrifuged (4.500 U/min; Mikro 22R, Hettich Instruments, Andreas Hettich AG, Bäch, Switzerland) for 30 minutes at about 8°C (Figure 1). The processed samples were ready for the LC-MS/MS analysis.

### 2.3. LC-MS/MS Settings

**Figure 2:**
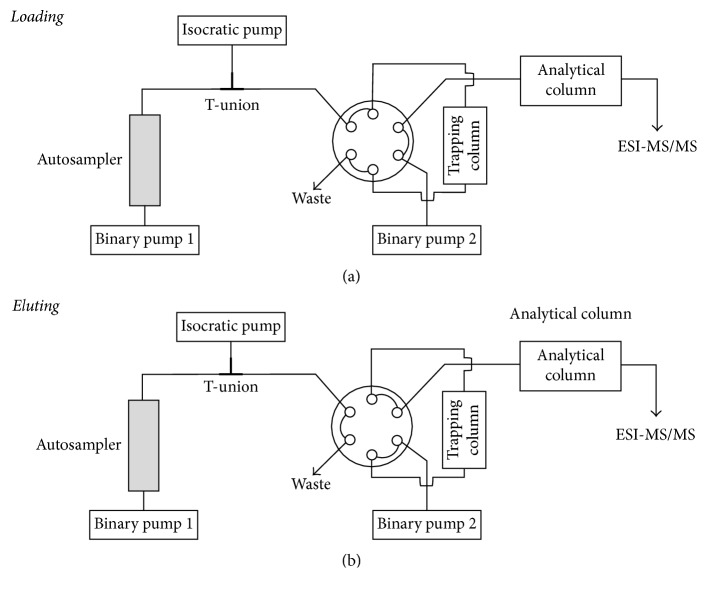
LC-MS/MS settings.

The prepared samples were placed into the autosampler (Dionex WPS-3000TSL Olten, Switzerland) which was set at 8°C. With a100 *µ*L syringe from the autosampler, 10 *µ*L of the prepared samples was injected into a 130 *µ*L loop. The solvent rack (Dionex SRD-3600, Olten, Switzerland) carried the mobile phase A (H_2_O + HCOOH (100 + 0.1, v + v)) and phase B (MeCN + HCOOH (100 + 0.1, v + v)). These mobile phases were delivered by three pumps (binary pump 1 (flow 0.350 mL/min) and isocratic pump 2 (flow 0.200 to 1.000 mL/min) (Dionex pump HPG-3200A, Olten, Switzerland), and binary pump 3 (Dionex pump ISO-3100A, Olten, Switzerland)) connected to a triple stage quadrupole mass spectrometer with linear ion-trap capability (3200 QTrap, Analyst Software Version 1.5.1, Applied Biosystems/MDS Sciex, Toronto, Canada) (Table 1). For the mass spectrometric detection, SRM scan mode (selective reaction monitoring) was used. SRM transitions and mass spectrometric conditions were as follows: transition: 253.1 → 182.2 (PHT) and 253.1 → 192.2 (PHT-D_10_); orifice (V): 36; collision energy (eV): 41 (PHT) and 51 (PHT-D_10_); and dwell time (msec): 100. Electrospray ionization was performed in positive ion mode for the analyte and the IS. The following instrument parameters for ionization were used: ion source voltage: 5000 volt, curtain gas: 25, gas 1: 40 and gas 2: 60; and the CAD gas was set to 5 (arbitrary units for the gas settings). As trapping column, a Phenomenex Gemini Polar column (2.0 × 10 mm, 5 *µ*m; Brechbühler AG, Schlieren, Switzerland) temperated to room temperature was used. The main column Phenomenex Synergy Polar RP column (2.0 × 50 mm; Brechbühler AG, Schlieren, Switzerland) was placed into the column oven (Cluzeau Info Labo CrocoCil) set on 50°C with a column thermostat (Dionex TCC-3100, Olten, Switzerland) including switching valve (Figure 2). This system was operated by Analyst Software (Version 1.5.1, AB Sciex, Toronto, Canada).

### 2.4. Validation of the LC-MS/MS Method according to ISO 17025/FDA Guidance for Industry

The validation was carried out according to ISO 17025/FDA Guidance for Industry including selectivity, sensitivity, accuracy, recovery of PHT, reproducibility and suitability of the calibration curves, stability of PHT, and matrix effects. The selectivity and sensitivity (absence of PHT) were verified by analyzing blank samples without PHT (extraction and matrix effects). For the accuracy, QCs and Cal samples were analyzed. The recovery of PHT was analyzed by measuring QCs at different levels. The reproducibility and suitability of the calibration curves was measured by a complete series of Cal 1 to Cal 8 (LC-MS/MS) analyses. The limit of detection (LOD) and the limit of quantification (LOQ) were analyzed using Cal 1 (LC-MS/MS 10 ng/mL PHT). The LOD was checked as a signal-to-noise (*S*/*N*) ratio of more than 4 : 1. The LOQ was considered as 5 times the response to a blank sample. The stability tests consisted of the freeze-thaw stability of PHT, which was determined after 3 freeze-thaw cycles. The short-term stability was analyzed by keeping the samples thawed at ambient temperature for at least 6 hours, frozen for at least 12 hours at −25°C ± 5°C, and again thawed, worked-up, and analyzed. Postpreparative stability was evaluated to determine whether an analytical run can be reinjected in the case of instrument failure and, furthermore, whether the preparation of a large number of samples could be done at once. Therefore, one of the validation runs was analyzed a second time after 7 days. The described criteria for Cal curves, QC, accuracy, and precision had to be met.

Matrix effects were analyzed by comparing the calibration curves generated with the three matrices aCSF, blood, and saliva. PHT microdialysis and blood samples from patients were analyzed to demonstrate the suitability of the method for biological samples from patients.

### 2.5. Comparison of the LC-MS/MS and the GC-MS Method

The LC-MS/MS method was evaluated and compared with the GC-MS method published by Hösli et al. [8] with regard to its analytical performance, appropriateness to analyze human biological samples, including corresponding extraction and cleanup procedures, and its validation according to ISO 17025/FDA Guidance for Industry.

The statistical data were calculated with Microsoft Excel and IBM SPSS Statistics 22. To compare the different matrices, a one-way ANOVA was calculated. The corresponding values were checked for significance by *t*-tests.

## 3. Results

### 3.1. Validation of the LC-MS/MS Method

**Figure 3:**
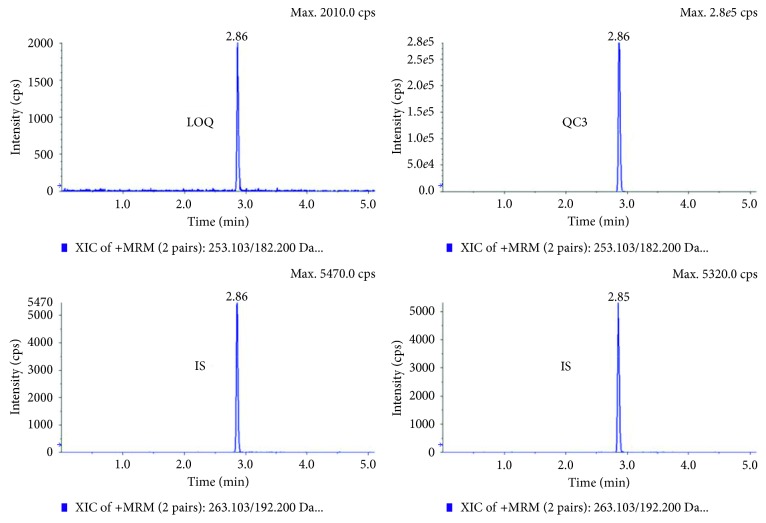
Chromatogram of phenytoin (illustrated for LOQ (10 ng/mL) and QC3 (400 ng/mL)) with PHT-D_10_ as IS (50 ng/mL).

The retention time (RT) for PHT and for PHT-D_10_ (IS) was about 2.8 min (Figure 3). The selectivity and sensitivity were checked; all blank samples were negative. The recovery of PHT after precipitation with HClO_4_ was 89.5% for QC1 (10 ng/mL) and 97.1% for QC3 (1600 ng/mL) compared to the amount found in unprepared samples (=100%). The LOD calculated as *S*/*N* ratio of 4 : 1 for this method in aCSF, saliva, and blood was set at <1 ng/mL. The LOQ calculated as 5 times the response/blank was 10 ng/mL PHT. For the accuracy, the Cal 1 to Cal 8 were assessed. The calibrator values showed min–max deviations of 1–8% for Cal 2 (20 ng/mL) to Cal 8 (2000 ng/mL) with 3% for Cal 1 (10 ng/mL). The calibration curves for all three matrices were linear. The regression coefficients (*r*^2^) of the three different matrices were *r*^2^_blood_ = 0.996 (*n* = 3), *r*^2^_dialysate_ = 0.997 (*n* = 6), and *r*^2^_saliva_ = 0.995 (*n* = 3). Reinjection after 7 days showed no difference in accuracy. The sample volume needed was 25 *µ*L. The sample preparation time was about 2 min per sample (6 hours for 182 samples). The run time for one LC-MS/MS analyses was 7 min.

### 3.2. Comparison of the LC-MS/MS with the GC-MS Method

After validation of the LC-MS/MS method, it was compared with the referred GC-MS method [8].  Table 2 shows the comparative results of the two methods for their analytical performance, appropriateness to analyze human biological samples, including corresponding extraction and cleanup procedures, and its validation according to ISO 17025/FDA Guidance for Industry (Table 2).

The selectivity and the sensitivity were met by both methods, and the recovery showed no differences (Table 2). But the accuracy differed between the two methods. The GC-MS method showed a higher variation at Cal 1 (20%) than the LC-MS/MS method (Cal 1 = 3%). As expected, the biggest difference in terms of analytical performance between the two methods was observed by the LOQ (GC-MS = 50 ng/mL; LC-MS/MS = 10 ng/mL) and LOD (GC-MS = 15 ng/mL; LC-MS/MS = <1 ng/mL) (Table 2).

**Figure 4:**
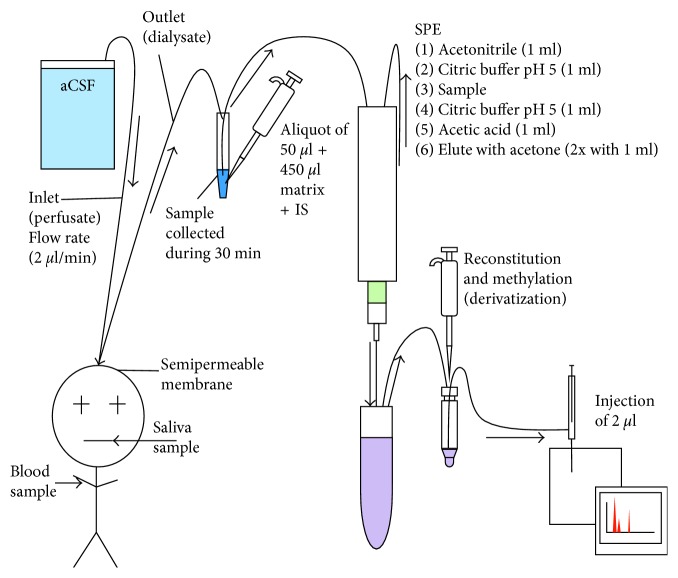
Sample preparation for the GC-MS analyses [8].

Both methods showed linear regression coefficients (*r*^2^) higher than 0.995 in all three different matrices for the PHT calibration curve. The calibration range of the LC-MS/MS (from 10 ng/mL to 2000 ng/mL) is twice as large as of the GC-MS (50 ng/mL to 1200 ng/mL). The stability of the samples after extraction and cleaning up was demonstrated for both methods. The sample preparation procedure is demonstrated in Figure 1 for LC-MS/MS and Figure 4 for GC-MS.

### 3.3. Comparison of the LC-MS/MS Method with a Formerly Established LC-MS/MS Method

Recently, a LC-MS/MS method has been developed which measures PHT in blood plasma or serum [10]. For the measurement of PHT in blood, the newly validated LC-MS/MS method can hence also be compared with this recently published study. The two methods show some similarities such as an identical IS (100 *µ*g/mL PHT-d10), similar sample volumes needed (25 *µ*L versus 20 *µ*L [10]), and a comparable retention time (2.8 min versus approximately 2.1 min [10]). Both methods showed linear regression coefficients (*r*^2^) higher than 0.99 in the blood matrix. The accuracy was similar as both studies showed deviations of <10%. With regard to the calibration range and the calibration solution, the two LC-MS/MS methods differ. While the LC-MS/MS method developed in this study showed a calibration range from 10 ng/mL to 2000 ng/mL, the calibration curve of the published LC-MS/MS method [10] ranged from 100 ng/mL to 4000 ng/mL. The calibration solution in the current study was the respective biological matrix (e.g., blood). In the published study [10], phosphate-buffered saline was used as the calibration solution.

## 4. Discussion

In this study, a LC-MS/MS method to measure PHT in different biological samples was successfully validated and compared with a similarly validated GC-MS method [8]. Overall, the LC-MS/MS method showed to be a more specific analytical method with a higher general performance (Table 2). The LC-MS/MS method needed less sample volume, less chemicals, and less analytical time and therefore resulted in less costs for the sample preparation.

Concerning the LOD, there was a huge difference between the two methods. The LOD of the LC-MS/MS method was 15 times better than the one of the GC-MS methods: the LOD of the LC-MS/MS method was <1 ng/mL compared to 15 ng/mL for the GC-MS method (increments by a factor of ten). Similarly, the difference in LOQ was 5 times lower in LC-MS/MS (10 ng/ml) compared to GC-MS (50 ng/mL). The LOQ for the LC-MS/MS could be set even lower than 10 ng/ml PHT (Cal 1). The FDA guidelines which claim a minimal reproducibility at the LOQ level of 20% were well below (deviation to target PHT amount: <8% in aCSF (*n* = 6), <4% in blood (*n* = 3), <9% in saliva (*n* = 3); accuracy: aCSF 103%, blood 101%, and saliva 106%). The LOQ of the GC-MS method and hence the lowest concentration level (Cal 1 at 50 ng/ml) of the calibration curve showed a deviation value of 19%. The LC-MS/MS method, in contrast, showed a value of only 3% deviation at the lowest Cal (10 ng/mL). This difference is of high importance, as samples with even lower concentrations could be reliably analyzed.

The calibration range (from 10 ng/mL to 2000 ng/mL) of the LC-MS/MS method was twice as large as of the GC-MS method (50 ng/mL to 1200 ng/mL). This indicates that the LC-MS/MS method is more powerful and effective over a larger range of concentration, since the linearity is given over a larger area (10 ng/mL–2000 ng/mL) compared to the GC-MS method (50 ng/mL–1200 ng/mL).

As IS, two different substances were used. MPPH as a structurally related compound was used for the GC-MS method. As IS for LC-MS/MS, deuterated PHT (PHT-D_10_) was used, which is the same molecule as PHT and differs only by the molecular mass (+1). All the physicochemical processes upon cleanup and analysis are identical or highly similar for PHT and PHT-D_10_. MMPH, however, could be chemically affected in a different way than PHT, which could lead to a systematic bias in a given situation [15].

Regarding the sample preparation procedure, the LC-MS/MS (Figure 1) showed an important advantage compared to the GC-MS method as it only needs 3 steps of sample preparation compared to 11 steps necessary for the GC-MS method including a solid-phase extraction (SPE) and derivatization with a more critical chemical trimethylsulfonium (TMSH) (Figure 4). This resulted in significant shortening of the overall analysis: Preparation of the samples before injection for GC-MS is about ten times more time consuming than for the LC-MS/MS. For the GC-MS method, researchers needed 5 hours to prepare 25 samples (5 samples/h), whereas for the LC-MS/MS method 182 samples were prepared in 6 hours (30.3 samples/h), which corresponds to 6 times the amount of prepared samples per hour compared to the GC-MS method.

From the exposure side, the volumes are much larger and the exposure to the chemicals are more prolonged with the GC-MS method compared to the sample cleanup for the LC-MS/MS method. Especially, the derivatization agent TMSH is critical to handle because of toxicity. The risk of serious and even irreversible effects through inhalation, skin contact, or eye exposure is well known. TMSH is also considered to be teratogenic. Therefore, the potential health risk for the laboratory staff handling the samples can be reduced by the LC-MS/MS method and the elimination of a safety critical agent.

The amount of biological samples needed for the GC-MS method (50 *µ*L) was twice as much as for the LC-MS/MS (25 *µ*L). The sample volume is a critical point for PK/PD studies, where, for example, by continuing dialysis from brain in neurosurgical patients only small volumes of samples per time point/period are available. For 50 *µ*L dialysate about 25 minutes collecting time is necessary at the usual flow rate of ∼2 *µ*L per minute [12, 16]. Therefore, not a requested specific time point, but a rather large time segment is represented which can influence the requested results. The reduced sample volume needed (25 *µ*L) for the LC-MS/MS analyses reduces the dialyses time needed per sample to about 15 minutes. The smaller the dialysis time, the more precise correlations of the respective tissue concentration with plasma/blood samples can be made.

Furthermore, LC-MS/MS also has the shorter run time. The time needed for 100 GC-MS analyses would be approximately 50 hours. The LC-MS/MS method, in contrast, needs only 11 hours and 40 minutes for 100 analyses. This is a time saving of more than 38 hours. While this may not be highly relevant for forensic purposes, for bedside and routine analyses (real-time) and PK/PD studies with larger numbers of samples, this factor is relevant. Also, when the time between taking a sample and the result needed is short, as it is in TDM to adjust subsequent dosing for PHT treatment, this time saving is crucial.

The costs for one way materials per sample was about 50% lower for the LC-MS/MS compared to the GC-MS method. Especially because no SPE device was needed. Also, the reduced work load for the laboratory technician must be considered as an imported cost factor.

Finally, the appropriateness of the method also depends on the biological matrix. Both methods can generally be used to measure PHT in blood and saliva, as the sample volume is less limiting. As mentioned before, however, for dialysates, the most difficult aspect is to get enough sample volume. Therefore, the LC-MS/MS method needing only half of the sample volume compared to the GC-MS method is more suited for microdialysate measurements. With respect to the LOD/LOQ, the LC-MS/MS method is also better suited for PK/PD studies, as it allows to include patients with low PHT dosages.

In addition, the newly established LC-MS/MS method was compared with a recently published LC-MS/MS method [10]. While this study measured PHT only in one body compartment (i.e., blood plasma or serum), the current LC-MS/MS method was developed and validated for the measurement of PHT in different body compartments (i.e., blood, saliva, and samples from brain tissue microdialysis often used in neurology and neurosurgery). The calibration range of the published LC-MS/MS method [10] (from 100 ng/mL to 4000 ng/mL) is appropriate for the measurement of PHT in blood plasma. As the PHT concentrations in brain tissue dialysates are much smaller than in blood plasma, the LC-MS/MS method of the current study was more appropriate for such samples, showing a lower calibration range from 10 ng/mL to 2000 ng/mL. Finally, as the aim of this study was to measure PHT in different biological matrices, a general substitute solution for blood plasma such as phosphate-buffered saline [10] could not be used. Instead, the fluid of the respective body compartment was used as calibration solution (e.g., artificial cerebrospinal fluid (aCSF) for the measurement of PHT in the brain tissue dialysates). This also eliminates a potential analytical bias due to matrix effects.

## 5. Conclusion

In this study, a LC-MS/MS method to measure PHT in different biological samples (i.e., human brain dialysate, blood, and saliva) was developed and validated under circumstances that ensured a high comparability with an established GC-MS method [8]. Overall, the study concludes that LC-MS/MS is not only better performing in human PHT concentration measuring or comparable drug PK/PD studies but is the only one to be used for bedside analysis. The time-consuming sample preparation and the long run time of the GC-MS method delay the result, which is critical in TDM. The higher sensitivity, the smaller needed sample volume, the better LOD/LOQ, the less time-consuming cleaningup and sample preparation procedure, and the shorter run time make the LC-MS/MS method the preferred analytical procedure.

## Figures and Tables

**Table 1:** Settings of the HPLC program.

Time (minutes)	Pump 1 (main column (MC))			Pumps 2 and 3 (trapping column (TC))			
	%*B*	Flow (*µ*L/min)	Comments	%*B*	Flow (*µ*L/min)	Flow pump 5 (H_2_O + 0.1% HCOOH) (*µ*L/min)	Switching valve
0	35	500	Start MS and pumps	50	300	800	TC → waste, MC → MS (loading)
0.5	35		Start gradient	50	300	800	TC → MC → MS (eluting)
0.6	↓			50	20	20	
1	97.5						
2	97.5						TC → waste, MC → MS
2.5	35			50	300	800	Reequilibration

**Table 2:** Comparison of the GC-MS [8] versus LC-MS/MS method.

Criterion	GC-MS	LC-MS/MS
Retention time	PHT 15.12 min, IS MPPH 16.15 min	PHT and PHT-D_10_ 2.8 min
Selectivity/sensitivity (absence of PHT)	Good peak differentiation and quantification of PHT. All blank samples were negative (no presence of PHT)	All blank samples were negative (no presence of PHT)
Recovery	94.1% for QC2 (100 ng/mL)	89.5% for QC1 (10 ng/mL)
	94.3% for QC5 (1000 ng/mL)	97.1% for QC3 (1600 ng/mL)
LOD (calculated as *S*/*N* ratio of 4 : 1)	15 ng/mL	<1 ng/mL
LOQ (calculated as 5 times the response/blank)	50 ng/mL	10 ng/mL
Accuracy	The calibrator values showed min–max percent deviations of 1–20% for Cal 1 (50 ng/mL) to Cal 6 (1200 ng/mL)	The calibrator values showed min–max percent deviations of 1–8% for Cal 1 (10 ng/mL) to Cal 8 (2000 ng/mL)
Regression coefficient, *r*^2^	*r* ^2^_blood_ = 0.998 (*n* = 2) *r*^2^_dialysate_ = 0.999 (*n* = 8) *r*^2^_saliva_ = 0.999 (*n* = 2)	*r* ^2^_blood_ = 0. 996 (*n* = 3) *r*^2^_dialysate_ = 0.997 (*n* = 6) *r*^2^_saliva_ = 0.995 (*n* = 3)
Calibration range	50–1200 ng/mL	10–2000 ng/mL
Run time per analysis	30 min	7 min
Injection volume of the sample	2.0 *µ*L	10 *µ*L
Sample preparation time	5 h for 25 samples	6 h for 182 samples
Stability of the processed samples	Dried extracts were stable for ≥4 weeks (min/max deviation 4%). No effect by reinjection and storage (33 h) on the autosampler	Reinjection after 7 days showed no difference in accuracy
Sample volume needed	50 *µ*L	25 *µ*L
